# Application of QSAR Approach to Assess the Effects of Organic Pollutants on Bacterial Virulence Factors

**DOI:** 10.3390/microorganisms11061375

**Published:** 2023-05-24

**Authors:** Roukaya Al Haj Ishak Al Ali, Leslie Mondamert, Jean-Marc Berjeaud, Joelle Jandry, Alexandre Crépin, Jérôme Labanowski

**Affiliations:** 1 Institute of Chemistry, Materials and Natural Resources of Poitiers, UMR CNRS 7285, University of Poitiers, 86000 Poitiers, France; roukaya.ishak@hotmail.com (R.A.H.I.A.A.); leslie.mondamert@univ-poitiers.fr (L.M.); 2 Ecology and Biology of Interactions, UMR CNRS 7267, University of Poitiers, 86000 Poitiers, France; 3 Faculty of Agronomy and Veterinary Sciences, Lebanese University, Dekwaneh, Lebanon

**Keywords:** QSAR, organic pollutants, virulence, *Escherichia coli* K12, *Pseudomonas aeruginosa* H103, *Salmonella enterica* serovar Typhimurium, pesticides, pharmaceutical compounds

## Abstract

The release of a wide variety of persistent chemical contaminants into wastewater has become a growing concern due to their potential health and environmental risks. While the toxic effects of these pollutants on aquatic organisms have been extensively studied, their impact on microbial pathogens and their virulence mechanisms remains largely unexplored. This research paper focuses on the identification and prioritization of chemical pollutants that increase bacterial pathogenicity, which is a public health concern. In order to predict how chemical compounds, such as pesticides and pharmaceuticals, would affect the virulence mechanisms of three bacterial strains (*Escherichia coli* K12, *Pseudomonas aeruginosa* H103, and *Salmonella enterica* serovar. Typhimurium), this study has developed quantitative structure–activity relationship (QSAR) models. The use of analysis of variance (ANOVA) functions assists in developing QSAR models based on the chemical structure of the compounds, to predict their effect on the growth and swarming behavior of the bacterial strains. The results showed an uncertainty in the created model, and that increases in virulence factors, including growth and motility of bacteria, after exposure to the studied compounds are possible to be predicted. These results could be more accurate if the interactions between groups of functions are included. For that, to make an accurate and universal model, it is essential to incorporate a larger number of compounds of similar and different structures.

## 1. Introduction

Over the past few decades, the release of numerous chemicals into wastewater has increased due to industrialization, urbanization, daily consumer products, and intensive agriculture [1,2,3]. These persistent contaminants, such as drugs, fragrances, preservatives, plasticizers, pesticides, polycyclic aromatic hydrocarbons (PAHs), phthalates (PAEs), polychlorinated bi-phenyls (PCBs), and others [4], pose serious health concerns to living organisms exposed to polluted waters even at low concentrations [5]. The toxicity of these compounds, including neurotoxicity, genotoxicity, endocrine disruption, and phytotoxicity, has been extensively studied [6,7,8,9] using bioassays with a wide range of organisms, such as fish, algae, daphnia, and micro-organisms [10,11,12,13]. However, the effects of pollutants on microorganisms are not limited to their toxicity, as microorganisms can sometimes use pollutants as a source of carbon or energy [14,15] or develop preservation mechanisms such as biofilm production, chelation, or efflux pumps that allow them to resist the effects of pollutants [16]. Therefore, studies suggest that exposure to chemical pollutants may also increase the pathogenicity of certain bacteria, thereby increasing the risk of infection [17]. For instance, some research shows that DEHP, through an unknown mechanism, increases the cytotoxicity of *Helicobacter pylori* [18]. The presence of triclosan appears to promote the proliferation of Gram-positive bacteria of the genera *Streptococcus*, *Enterococcus*, and *Staphylococcus* [19], including pathogenic species. This compound can also promote the production of biofilms, which are considered a virulence factor in *Pseudomonas aeruginosa* [20].

The identification of chemical determinants involved in bacterial virulence is recognized as a public health issue [21]. However, the acquisition of complete data for all pollutants and microorganisms is complex due to the colossal number of chemical compounds and pathogenic or potentially pathogenic microorganisms. Therefore, the development of efficient strategies for the identification and prioritization of pollutants and their effects on pathogenicity is essential for public health management. Among them, the use of in silico prediction, which was promoted by the implementation of REACH, has become a promising alternative [22]. In silico models have been widely used in medicine, pharmaceuticals, and various industries to predict the toxicity and safety of chemicals, including quantitative structure–activity relationship (QSAR) and quantitative structure–property relationship (QSPR) models [23,24,25]. Different studies have used multiple linear regression (MLR) and partial least square (PLS) methodologies to investigate the correlation between the retention factor and a number of molecular descriptors for a series of phosphoramidic acid derivatives [26]. The purpose of this article was to use a QSAR approach based on the chemical compound structure to assess, in silico, how certain virulence parameters of three bacterial models were affected. Seventeen compounds were individually tested to create a database for constructing predictive models. Therefore, 3 bacterial strains were exposed to strains to 17 different chemical compounds, including pesticides and pharmaceuticals, to investigate their effects on growth and swarming behavior. The resulting dataset was used to develop quantitative structure–activity relationship (QSAR) models through analysis of variance (ANOVA) functions. Furthermore, the expression of several virulence genes was measured for five of the compounds to further understand the potential impact of pollutants on bacterial virulence. This work thus aims to contribute to the knowledge base in the search for potentially relevant tools/methods to screen and identify, a priori, the compounds that pose the most risk for environmental and/or human health.

## 2. Materials and Methods

### 2.1. Model Chemical Compounds

A total of 17 chemical compounds—10 pesticides and 7 pharmaceutical compounds—were selected for this study. The structures of these compounds are presented in the Supplementary Materials (Tables S1 and S2), and their physicochemical structures are presented in Table S3.

### 2.2. Microbial Activity Tests

#### 2.2.1. Bacterial Strains and Culture Conditions

Three bacterial strains were studied: *E. coli* K12, from the EBI laboratory collection, *Pseudomonas aeruginosa* H103, which was isolated from patients with cystic fibrosis [27], and *Salmonella enterica* serovar. Typhimurium (hereafter: *S.* Typhimurium), which was purchased from ATCC (American Type Culture Collection): ATCC^®^ 14028™ from Washington, DC, USA. All of the strains were maintained in a Lysogeny broth medium (LB) (1% Tryptone, 0.5% yeast extract, and 1% NaCl, BIOKAR Diagnostics, Allonne, France) containing 30% glycerol (*v*/*v*) (Sigma Chemical, St. Louis, MI, USA) at −80 °C. The bacteria were pre-cultured by transferring a typical colony onto a Petri dish containing LB agar (1.5% agar type E), followed by incubation at 37 °C for 24 h.

#### 2.2.2. Bacteria Growth Test

A volume of 200 μL of a solution of a single chemical compound (pesticide or pharmaceutical) was diluted in 4 mL of LB broth to prepare a serial dilution of 7 concentrations (950, 190, 38, 7, 1, 0.3, and 0.06 μg/L) in polypropylene conical tubes (15 mL, SARSTED, Singapore). The three bacteria strains were diluted in LB broth to yield an inoculum of approximately 10^6^ CFU/mL, determined by optical density at 600 nm using a JENWAY 6320D spectrophotometer. Bacterial inoculum was then inoculated into the test solutions (950, 190, 38, 7, 1, 0.3, and 0.06 μg/L) with 5% methanol on a 96-well plate (Nunclon™ Delta Surface, Thermo Scientific, Waltham, MA, USA). Growth was measured after 24 h of incubation at 37 °C by optical density at 595 nm using an Infinite M Plex microplate reader (TECAN, Männedorf, Switzerland). The percentage of growth was calculated relative to the control cultures of each strain (5% MeOH) after 24 h of incubation. All measurements were performed in triplicate. The result of each concentration was statically analyzed against the control (5% MeOH) to determine its significance, using one-way ANOVA followed by Dunnett’s post-hoc test.

#### 2.2.3. Swarming Assay

The swarming motility assay was performed for the three bacteria strains by stab-inoculating the center of LB solid medium (0.6% agar) with the bacteria suspension (control, i.e., in 5% MeOH, 0.3, and 0.06 μg/L) at room temperature. Plates were covered and held for 10 min at room temperature, then placed upside-down in the incubator at 37 °C for 24 h (*P*. *aeruginosa* H103) or 48 h (*E. coli* K12 and *S*. Typhimurium).

#### 2.2.4. Relative Expression of Virulence Genes

*P*. *aeruginosa* H103 and *S.* Typhimurium were cultivated in test solutions at 37 °C in 4 mL of LB broth for 24 h. For each bacterium, two cultures for one chemical compound (pesticide or pharmaceutical) were prepared, one at 0.3 μg/L and another one at 0.06 μg/L, which represented the real concentrations usually found in the aquatic environment. Then, cultures were centrifuged at 5000× *g* for 10 min at 4 °C. After discarding the supernatant and removing any remaining media by aspiration, bacteria pellets were kept at −80 °C for mRNA extraction.

##### RNA Extraction

An RNeasy Mini Kit (QIAGEN, Paris, France) was used to extract the mRNA from the *P. aeruginosa* H103 and *S.* Typhimurium cultures. The bacteria envelopes were destructed by incubation at 37 °C for 10 min after adding 8 μL of the lysozyme (50 mg/mL). The mRNA samples were then processed by following the manufacturer’s protocol.

##### Real-Time PCR

The GoScriptTM Reverse Transcription kit (Promega) was used for the cDNA synthesis, according to the manufacturer’s recommendations. The prepared mixture, containing 4 μg of DNase-treated RNA and 1 μg of Primer oligo dT/Random primer (1:1), was heated at 70 °C for 5 min and then chilled on ice for 5 min. An amount of 15 μL of RT–PCR reaction mixture was then added to each sample. The cycle conditions were as follows: 5 min at 25 °C, 60 min at 42 °C, and then 15 min at 70 °C with RT inactivation. The resulting cDNA samples were set at −20 °C.

##### Quantitative PCR

The *P. aeruginosa* H103 sequences of *algD* (resistance to the immune system), *exoS* (acute infection), *lasI* (formation of biofilm), and *oprL* (outer membrane protein, reference) genes, together with the *S.* Typhimurium sequences of *invA* (invading the epithelial cells), *stn* (production of enterotoxin), and *rss16S* (reference) genes, were quantified when using the primers (the sequences are presented in Table S4) that were purchased from the Eurogentec partnership. The quantitative PCR (qPCR) was designed to amplify the bacteria genes when using Takyon™ SYBR^®^ 2× qPCR MasterMix Blue (Eurogentec, Liege, Belgium) and the LightCycler^®^ 480 system (Roche Applied Science, Penzberg, Germany). The reaction mixture contained 2.4 μL of UPW, 5 μL of 2× Takyon™ MasterMix, 0.3 μL of each primer (10 μM), and 2 μL of the diluted cDNA template, to form a final volume of 10 μL. Negative control was performed to examine any genomic DNA contamination. The qPCR conditions were as follows: initial denaturation at 95 °C for 5 min, followed by 45 cycles at 95 °C for 10 s, 57 °C for 10 s (for all of the studied genes except *oprL* and *rss16S*, for which the specific temperatures were 51 °C and 60 °C, respectively), and 72 °C for 10 s. A melting curve analysis ranging from 65 to 95 °C was used, to specify the amplicon for each primer pair.

The determination of gene expressions was performed in triplicate. The number of gene copies was calculated by using a constructed standard curve. The quantitative variation between the replicates was evaluated when using the 2^−∆∆CT^ relative quantification method [28]. The relative expressions of the *algD*, *lasI*, and *exoS* genes were normalized to the *oprL* gene, whereas *stn* and *invA* were normalized to the rss16S gene.

### 2.3. Validation of Exposure Conditions to Organic Pollutants

Many organic pollutants, especially pesticides, are naturally not very (or not at all) soluble in water. Therefore, they are preferentially solubilized and stored in less polar organic solvents (e.g., alcohol, DMSO, etc.) or even in non-polar organic solvents (e.g., cyclohexane or pentane). However, these solvents are also organic compounds, and their presence can interfere with—or even bias—the effect of the pollutant.

Bacteria growth tests were performed for the three studied strains with LB supplemented in MeOH to confirm any effect on bacterial growth. These tests showed that bacteria are able to grow, without significant perturbation, in the presence of up to 5% of MeOH concentration (Figure S1). Thus, this value was chosen for experiments.

### 2.4. Structure–Activity Models

QSAR Models Were Built Using ANOVA Function under XLstat Software (V 2022.4.5).

## 3. Results and Discussion

### 3.1. Collection of Experimental Data

#### 3.1.1. Effect of Selected Organic Pollutants on Bacterial Growth

The individual effect of 17 organic pollutants (10 pesticides and 7 pharmaceutical compounds) on the growth of *E. coli* K12, *P. aeruginosa* H103, and *S.* Typhimurium were tested. Growth tests were carried out after contact of the bacteria with different concentrations (from 0.06 to 950 µg/L) of each compound.

(a) Organochlorine pesticides (α-HCH, β-HCH, Lindane, 2,4′-DDD, Endosulfan)

**Figure 1:**
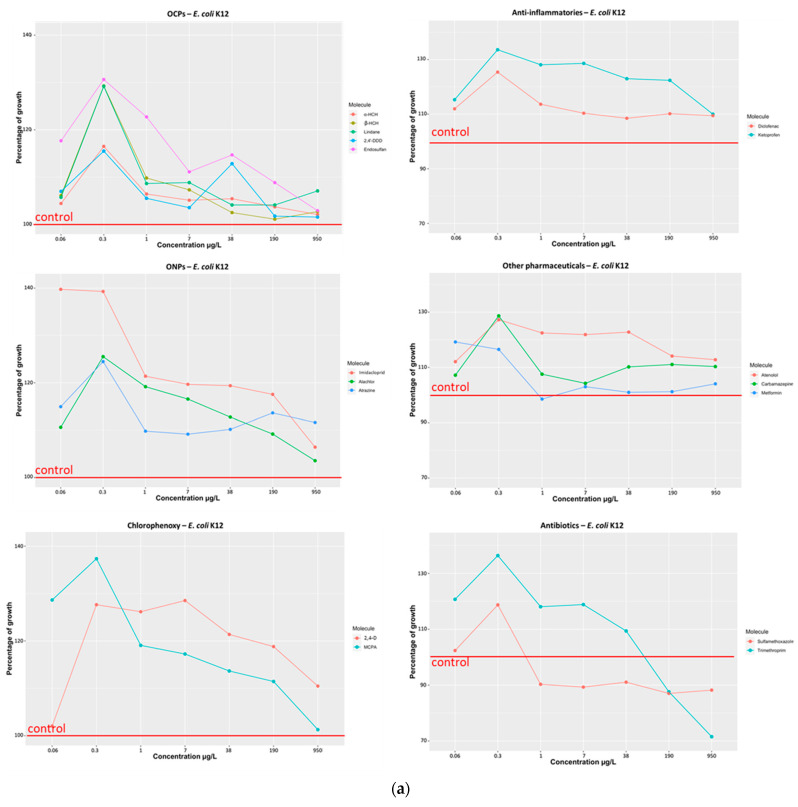

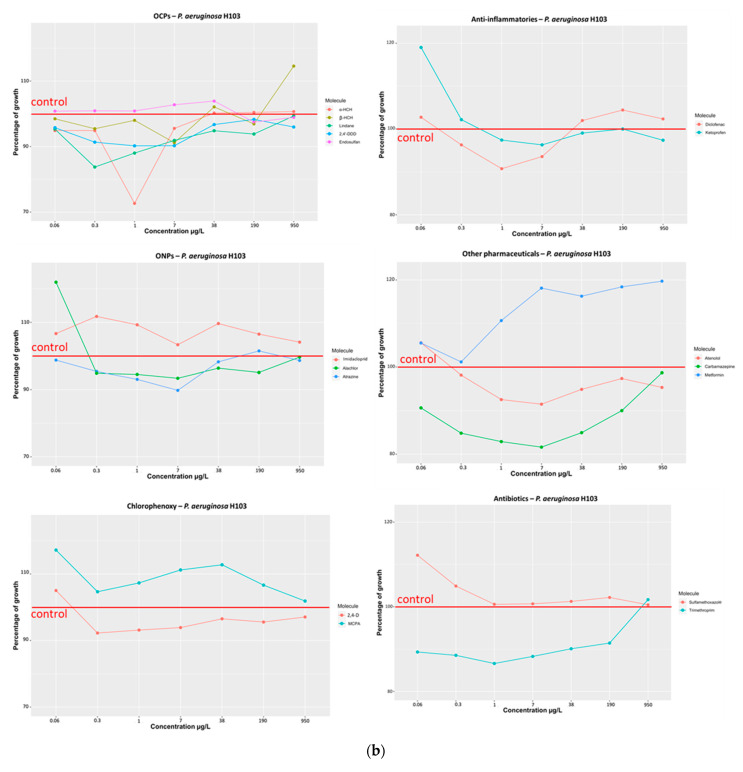

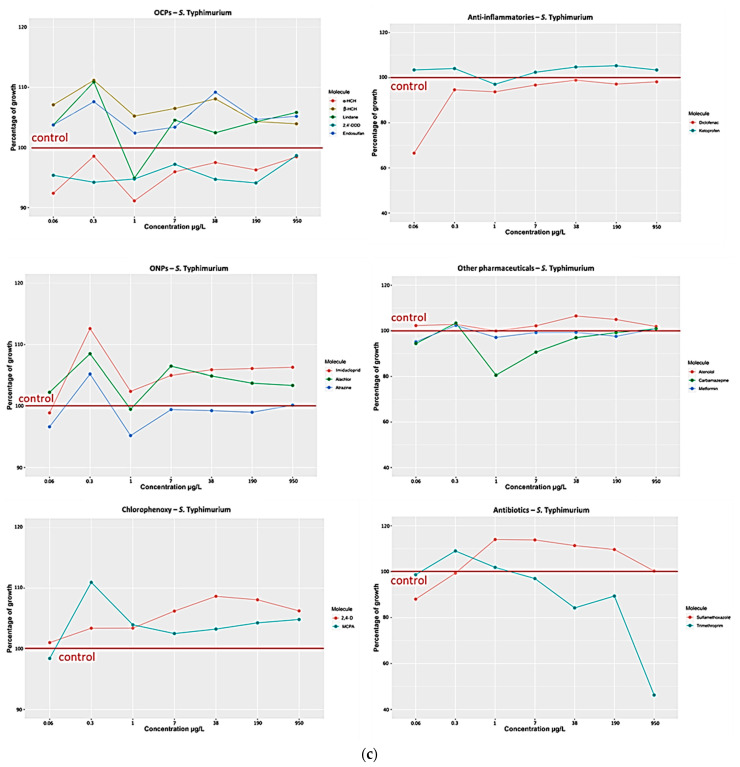
(**a**) *E. coli* K12 growth in the presence of individual pesticides or pharmaceutical compounds at concentrations ranging from 0.06 to 950 μg/L. (**b**) *P. aeruginosa* H103 growth in the presence of individual pesticides or pharmaceutical compounds at concentrations ranging from 0.06 to 950 μg/L. (**c**) *S.* Typhimurium growth in the presence of individual pesticides or pharmaceutical compounds at concentrations ranging from 0.06 to 950 μg/L.

The exposure to various organochlorine pesticides showed that all these compounds significantly increased the growth of *E. coli* K12 (Figure 1a). Endosulfan had the greatest effect on this bacterium, increasing its growth by up to 30% (at 0.06 and 0.3 µg/L). Lindane also increased the growth of *E. coli* K12 by up to 30% (at 0.3 µg/L). In general, exposure to organochlorines induced a greater increase in growth at the exposure concentration of 0.3 µg/L. The effect was less significant and decreased between 1 and 950 µg/L. Similarly, the effect on growth was also less significant at the lowest concentration (i.e., 0.06 µg/L). *P. aeruginosa* H103 reacted differently to organochlorine exposure (Figure 1b), as only one compound (β-HCH) had an increasing effect on its growth (up to 10%) at only one concentration (950 µg/L). One compound had no effect (endosulfan), and two compounds (lindane, 2,4′-DDD) decreased growth, especially at 0.3 µg/L. Except for 1 g/L, where the growth of *P. aeruginosa* H103 was reduced by over 30%, α-HCH had essentially no impact on the growth of this strain. Several compounds also had a growth-enhancing effect on *S.* Typhimurium (Figure 1c): lindane, endosulfan, and β-HCH, especially at 0.3 µg/L (nb. 38 µg/L for endosulfan). Their effect was generally less significant at higher concentrations (1–950 µg/L) and at the lowest concentration studied (0.06 µg/L). However, α-HCH and 2,4′-DDD behaved differently from the other organochlorines, as they reduced growth by up to 5%;

(b) Organonitrogen/chlorine compounds (Imidacloprid, Atrazine, Alachlor)

The exposure to organonitrogen/chlorine compounds (i.e., ONPs on figures) also increased the growth of *E. coli* K12 (Figure 1a). The effect was the highest at low concentrations for imidacloprid and at 0.3 µg/L for alachlor and atrazine. For *P. aeruginosa* H103 (Figure 1b), each compound caused a different effect. Imidacloprid increased growth by about 5 to 10% for all concentrations. In contrast, alachlor and atrazine reduced their growth at concentrations between 0.3 and 38 µg/L, but had little/no effect above that. At 0.06 µg/L, atrazine had no effect, while alachlor increased the growth of *P. aeruginosa* by more than 20%. Alachlor and imidacloprid increased the growth of *S.* Typhimurium (Figure 1c), especially at 0.3 µg/L and 7 to 950 µg/L;

(c) Chlorophenoxy compounds (MCPA and 2,4-D)

Both compounds promoted the growth of *E. coli* K12 (Figure 1a). MCPA had a maximum effect at 0.3 µg/L and 0.06 µg/L, and then had a smaller effect at higher concentrations. 2,4-D had a maximum effect between 0.3 and 7 µg/L, but virtually no effect at 0.06 µg/L. These compounds had different effects on *P. aeruginosa* H103 (Figure 1b). MCPA increased its growth by about 2–18%, while 2,4-D slightly decreased it (~2–8%) between 0.3 and 950 µg/L. Chlorophenoxy compounds had little/no effect at 0.06 µg/L on *S.* Typhimurium (Figure 1c) growth, but increased it at higher concentrations;

(d) Anti-inflammatory compounds (Ketoprofen and Diclofenac)

The exposure to anti-inflammatory compounds significantly increased the growth of *E. coli* K12 (Figure 1a). The results showed that the effect was 5 to 20% greater for ketoprofen than for diclofenac. On the contrary, these compounds had much less effect on *P. aeruginosa* H103 (Figure 1b) and *S.* Typhimurium (Figure 1c). Only ketoprofen significantly increased *P. aeruginosa* H103 growth at 0.06 g/L, while only diclofenac significantly decreased *P. aeruginosa* H103 growth at 1 g/L and *S*. Typhimurium growth at 0.06 g/L;

(e) Other pharmaceuticals (Atenolol, Carbamazepine, Metformin)

Other drug-like compounds were also examined to study their effect on bacterial growth. Atenolol and carbamazepine increased *E. coli* K12 growth (Figure 1a). This effect was observed with atenolol over the entire concentration range and with carbamazepine mainly at 0.3 µg/L. The exposure to metformin also increased *E. coli* K12 growth at the lowest concentrations tested (0.06 and 0.3 µg/L). *P. aeruginosa* H103 was also affected by these three compounds (Figure 1b). Its growth decreased notably in the presence of carbamazepine and atenolol (except at 0.06 µg/L). Metformin had no effect at low concentrations (0.06 and 0.3 µg/L), but significantly increased the growth of *P. aeruginosa* H103 between 1 and 950 µg/L. Atenolol and metformin had no effect on *S.* Typhimurium (Figure 1c). Only carbamazepine (only at 1 and 7 µg/L) led to a 10 to 20% reduction in the growth of this bacterium;

(f) Antibiotics (Sulfamethoxazole, Trimethoprim)

Both antibiotics promoted the growth of *E. coli* K12 (Figure 1a) at exposure concentrations up to 0.3 µg/L for sulfamethoxazole and 38 µg/L for trimethoprim. At higher concentrations, these antibiotics reduced the growth of this bacterium. Such antibiotics are used as a treatment for bladder infections caused by *E. coli*. The growth of *P. aeruginosa* H103 (Figure 1b) and of *S.* Typhimurium (Figure 1c) were also strongly reduced by trimethoprim. On the other hand, sulfamethoxazole seemed to favor the growth of *P. aeruginosa* H103 at low concentrations and of *S.* Typhimurium at high concentrations.

All these findings support the idea that different chemical compounds can have an effect on the growth of pathogenic bacteria. It is well known that some bacteria can take advantage of low pesticide concentrations to use them as an energy source [29,30,31,32]. Microbes also have the ability to degrade contaminants to multiply [33,34], even if these contaminants are toxic to other strains [35]. Some bacteria are or become resistant to toxic compounds—notably antibiotics—allowing them to grow in their presence [36,37,38,39]. Thus, most antibiotics can contribute to the growth of bacteria at concentrations below the minimal inhibitory concentration [40]. However, not all compounds are beneficial to bacteria. For example, one study showed that increasing pesticide concentrations had little or no effect on the growth of *E. coli* [41]. It has also been noted that some herbicides, insecticides, and fungicides are able to promote changes in the microbial community, whether in aquatic or terrestrial environments [42,43].

Thus, the growth of bacteria depends on the concentration of pollutant residues and on the tested strain.

#### 3.1.2. Effect of Selected Organic Pollutants on Bacterial Swarming

The interaction between bacterial cells enables the simultaneous growth and rapid spread of bacterial colonies on the surface. This pattern, which is known as “swarming”, directs the new cells to the edge of the colony and contributes to host infection. To test the effect of chemical compounds on swarming abilities, exposure experiments were performed on the three model strains and then their mobility was assessed. Two exposure concentrations were tested: low (0.06 µg/L) and moderate (0.3 µg/L).

**Figure 2:**
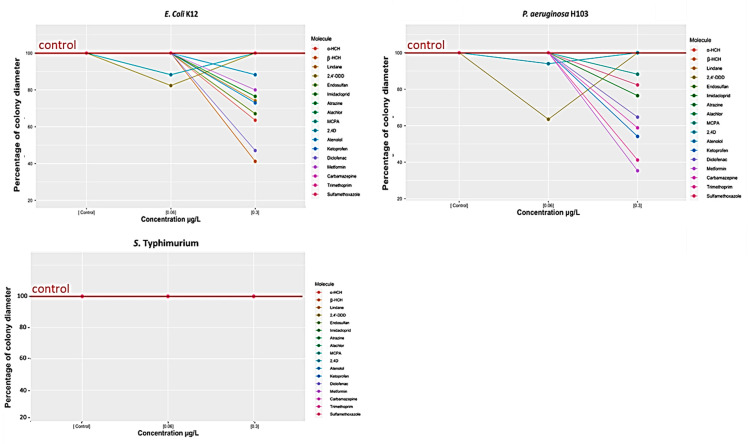
*E. coli* K12, *P. aeruginosa* H103, and *S.* Typhimurium swarming tests in the presence of individual pesticides or pharmaceutical compounds at concentrations of 0.06 and 0.3 μg/L.

The results showed that exposure to 0.06 µg/L (Figure 2) had little effect on bacterial swarming. Indeed, the growth diameters were the same between the bacteria exposed to the compounds and the controls corresponding to the three strains. Only 2,4-D and 2,4’-DDD caused a slight decrease in the swarming of *E. coli* K12 (12 and 17.6%, respectively) and *P. aeruginosa* H103 (6 and 35%, respectively). No compound influenced *S.* Typhimurium swarming.

In contrast, at a higher concentration (0.3 μg/L), more compounds had an effect and disrupted the swarming of *E. coli* K12 and *P. aeruginosa* H103, but the swarming of *S.* Typhimurium was still not influenced. Thus, 9 compounds (of the 17 studied) decreased *E. coli* K12 swarming. In addition, β-HCH and diclofenac showed a significant decrease, by 59% and 53%, respectively. Ten compounds decreased *P. aeruginosa* H103 swarming, in particular, metformin and trimethoprim (65% and 59%, respectively).

Thus, these findings show that both the concentration and nature of compounds influence the swarming and, hence, the potential virulence, especially for *P. aeruginosa* H103 and *E. coli* K12. It is worth noting that the low concentrations maintained this virulence factor, while exposure to high concentrations attenuated it.

**Figure 3:**
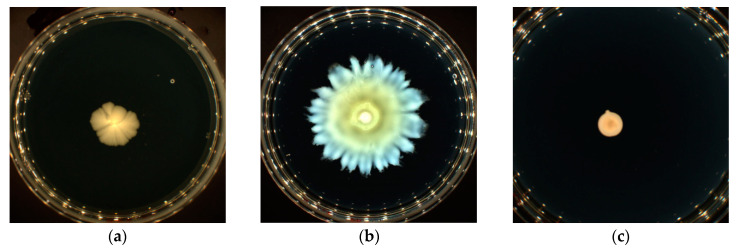
(**a**) E. coli K12 [MCPA] = 0.06 μg/L. (**b**) *P. aeruginosa* H103 [Alachlor] = 0.3 μg/L. (**c**) *S.* Typhimurium [Alachlor] = 0.3 μg/L. Photographs were taken and evaluated using Scan 1200^R^ (Interscience).

The swarming tests also showed that each bacterium displayed a specific colony pattern while swarming. For example, *E. coli* K12 adopted the simplest pattern, called “featureless”, that dilates equally in all directions with a transparent edge (Figure 3a). This pattern consists of a monolayer characterized by a high and uniform density [44]. On the contrary, *P. aeruginosa* H103 had a different pattern: dendritic swarming (Figure 3b). These dendrites are a thin region of colonization that expand from the inoculation point [45,46]. *S.* Typhimurium was characterized by non-swarming cells that were unable to emerge from the center colony to the surface (Figure 3c).

#### 3.1.3. Relative Expression of Virulence Genes after Exposure to Five Selected Organic Pollutants

Three genes associated with the virulence of *P. aeruginosa* H103—*algD* (resistance to the immune system), *exoS* (acute infection), *lasI* (formation of biofilm)—and two others for *S.* Typhimurium—*invA* (invading epithelia’s cells) and *stn* (production of enterotoxin)—were established in this study. These virulent genes were measured in cultures exposed to three pesticides (α-HCH, 2,4′-DDD, and atrazine) or two pharmaceutical compounds (diclofenac and metformin). Only a small number of concentrations were studied due to the experimental costs of qPCR.

**Figure 4:**
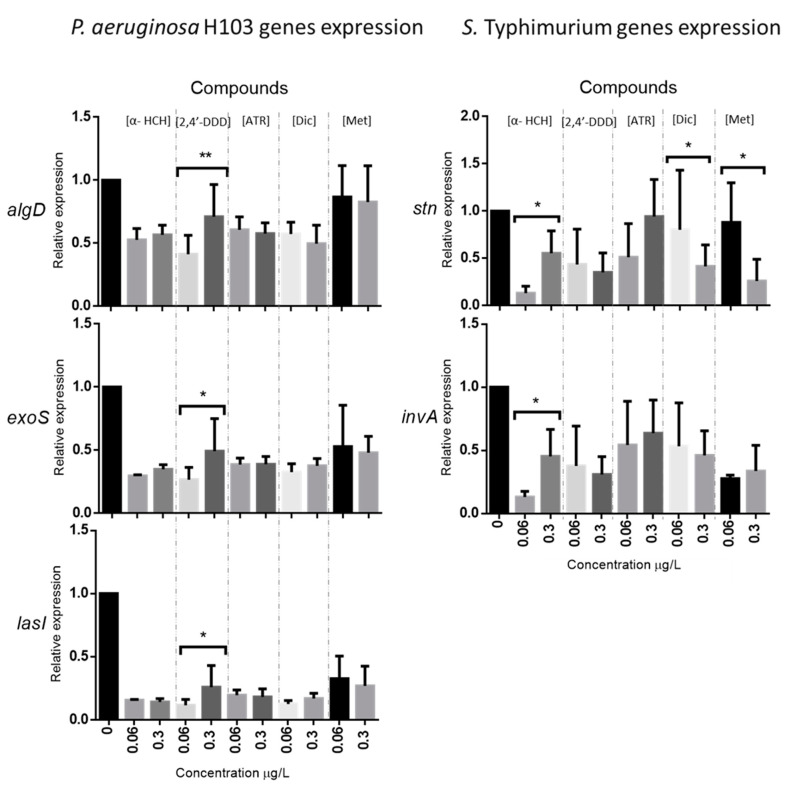
Relative expression of *algD*, *exoS* and *lasI* genes of *P. aeruginosa* H103 and *stn* and *invA* genes of *S.* Typhimurium after exposure of these strains to three pesticides and two pharmaceutical compounds. The relative expression of the virulence genes was compared to their reference genes (*oprL* and *rss16S* for *P. aeruginosa* H103 and *S.* Typhimurium, respectively). Results were subjected to unpaired (independent) parametric *t*-test (GraphPad Prism version 6.01 for Windows, GraphPad Software, La Jolla, CA, USA, www.graphpad.com, accessed on 21 May 2023). For qPCR tests, statistical analyses were performed on ΔCt values. All test results were significant when *p*-values were < 0.05 (* *p* < 0.05; ** *p* < 0.01). [ATR] = atrazine; [Dic] = diclofenac; [Met] = metformin.

At 0.06 µg/L, the results (Figure 4) highlighted that the relative expression of the two virulence genes (*exoS* and *lasI*) of *P. aeruginosa* H103 were strongly under-expressed in the presence of each of the pollutants. Indeed, results showed that exposure of *P. aeruginosa* H103 to pollutants led to a decrease of 75–85% for the *lasI* gene and 50–75% for the *exoS* gene. The *algD* gene was also under-expressed—albeit to a lesser extent (20–50%)—for all compounds, and even without significant variation for metformin. The two genes (*invA* and *stn*) of *S.* Typhimurium were also under-expressed, but in different ways for each compound. Thus, the results showed a large decrease for α-HCH and a decrease for 2,4′-DDD and atrazine. Both pharmaceutical compounds (diclofenac and metformin) generated more variable effects on *S.* Typhimurium genes. Metformin also decreased *invA* but had no effect on *stn*. Diclofenac had little effect overall, but uncertainties were high.

At a higher concentration (0.3 μg/L) (Figure 4), no other differences in effect were observed for the *algD*, *lasI*, and *exoS* genes of *P. aeruginosa* H103; only their expression seemed to increase with 2,4′-DDD. On the other hand, a higher concentration led to several changes in the expression of the *S.* Typhimurium genes. Thus, the exposure to 0.3 µg/L of α-HCH significantly (×3) increased the expression of the *stn* and *invA* genes. In contrast, the exposure to 0.3 µg/L of diclofenac or metformin caused a significant decrease in the *stn* gene.

There are few data of this kind available for the compounds studied (note: for the analyzed genes), but our results are consistent with a multitude of observations documented in the scientific literature for other organic compounds. Virulence gene expression linked to adhesion, biofilm formation, efflux pumps, and other defense mechanisms can be inhibited, repressed [47,48,49,50], or overexpressed [19,20,51,52] by organic compounds.

### 3.2. Structure–Activity Modelling

The influence of the chemical structure of a compound on its biological activity is a well-known notion. Thus, numerous mathematical approaches (regression or classification) have been developed to build equations/models relating to structure and activity (QSAR models). Each model is based on descriptors characteristic of the compounds and their effects. In this study, two types of descriptors were studied: global physicochemical descriptors and functional group descriptors.

#### 3.2.1. Structure–Properties–Activities Relationship

There is a wide range of descriptors that can be used to describe the chemical structure that allow particular properties of compounds to be linked to their effects or behavior. Physicochemical properties (molecular weight, logP, pKa, solubility, etc.) are among the most-used descriptors in ecotoxicology. Indeed, the octanol/water partition coefficient, log K_ow_, and the solubility in water are generally significant descriptors of the accumulation of compounds in cell membranes [53].

**Figure 5:**
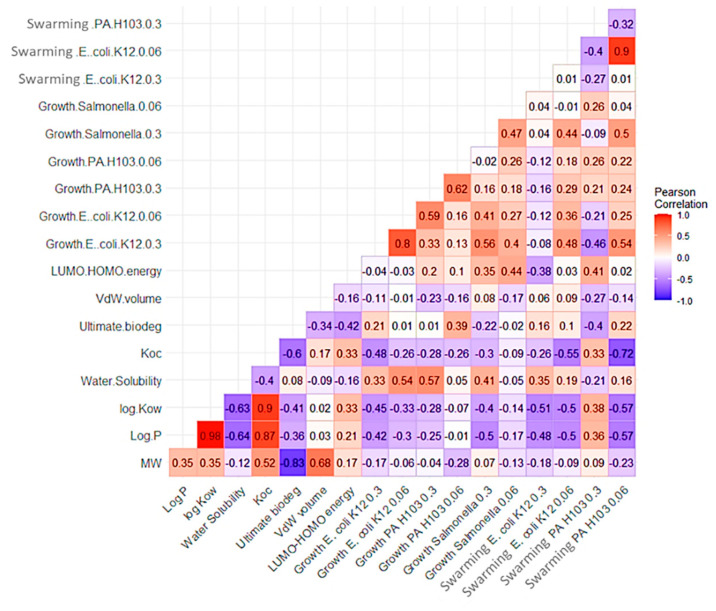
Pearson correlation matrix between physicochemical descriptors of the compounds and bacterial growth and swarming results.

Relationships between bacterial growth or swarming of *E. coli* K12, *P. aeruginosa* H103, and *S.* Typhimurium and the classical physicochemical descriptors were sought from the data obtained for the 15 model compounds. However, no linear relationship was observed, as shown by the Pearson correlation matrix (Figure 5).

#### 3.2.2. Structure–Activity Relationship (SAR) Model

Modelling based on the chemical structure has also been tested to predict the effect of compounds on bacterial growth or swarming. Indeed, QSAR models can be created from the knowledge of molecular groups or fragments. This type of model uses chemoinformatic algorithms to map and identify the functional groups present in organic molecules. They are increasingly used in healthcare and industry to study the effect of molecular fragments of interest in relation to variation in biological response, and/or to predict the physicochemical or biological properties of compounds based on knowledge of their chemical structure.

(a) Determination of chemical structure descriptors

For the construction of models, the chemical structure of compounds must be coded into mathematical variables. For this, the Organic Function Group “nested” algorithm (ECHA QSAR toolbox) was used to break the structure into fragments and count occurrences. The “nested” algorithm was preferred to the “general” algorithm because it does not show the functional groups which were nested in the larger ones (i.e., the aldehyde group is nested in a carboxylic group).

**Figure 6:**
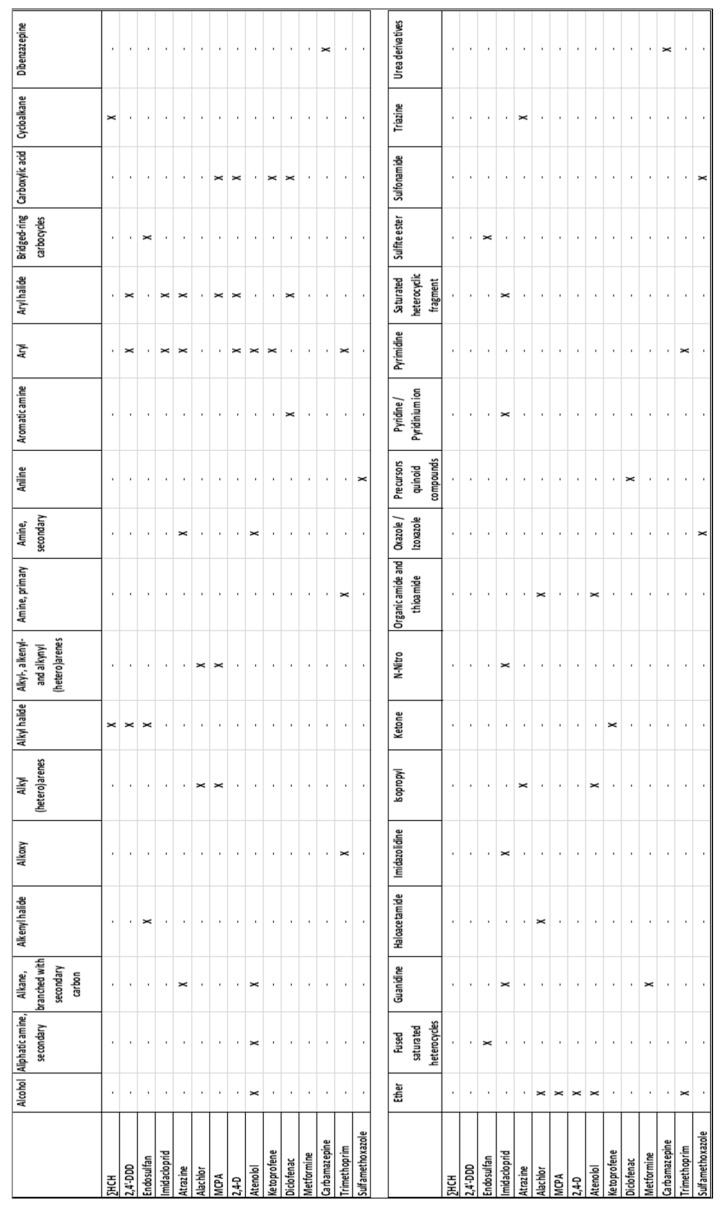
Matrix of organic functions defining the structure of compounds (n.b., x = function is present in the compound).

Figure 5 shows the set of functions identified by the algorithm in the 15 model compounds studied (n.b., the three HCH isomers were grouped into one compound, ∑HCH). In total, 38 main function groups were identified, but some of them were only present in the structure of one compound or were redundant. Therefore, only 11 functions, each represented at least twice in the whole set of compounds, were kept when building the models (Figure 6).

It is important to keep in mind that the limited number of compounds studied limits the representativeness of some functions (see Table 1). Thus, the approach proposed aims to show the feasibility of the scientific approach and does not claim to build the ideal model;

(b) SAR model for bacteria growth

**Figure 7:**
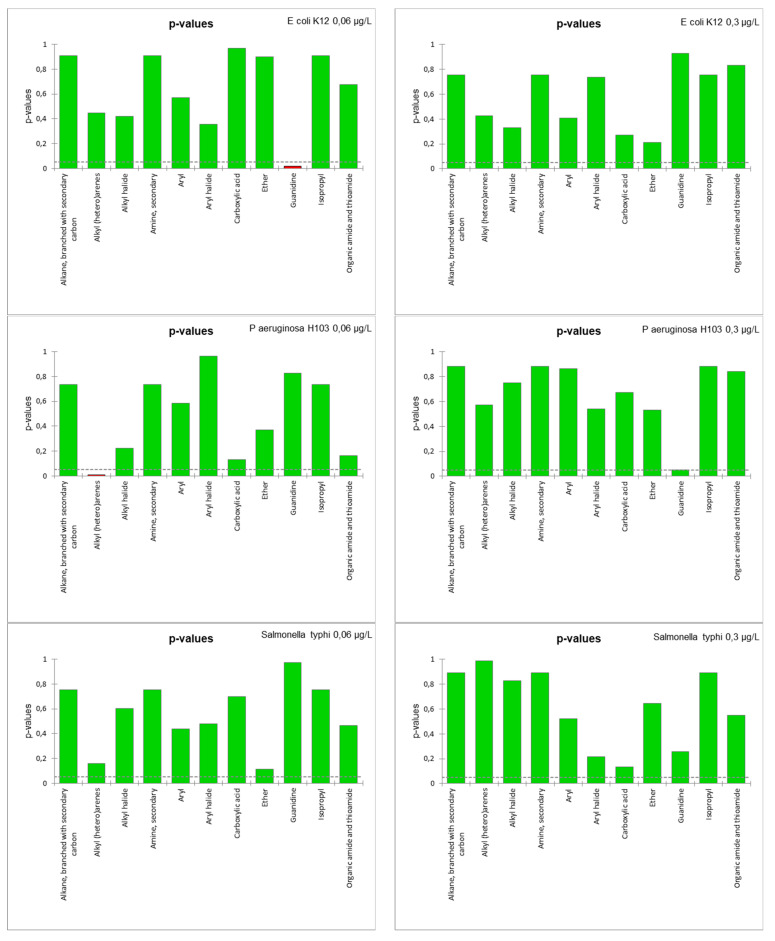
*p*-values of the Fisher F-test for the functional groups used as descriptors of growth data.

In order to define the most relevant descriptors, a Fisher F-test was performed to determine the ability of functional groups (nominal variables) to characterize the “growth” and “swarming” (quantitative variables). The results of this test for “growth” (Figure 7) showed that the *p*-values of the different groups were well above the significance level (n.b., defined at 95%) for the growth data, regardless of the strain or exposure concentration. Only the guanidine and alkyl (hetero)arene groups appeared as potentially significant descriptors of growth of *E. coli* K12 at 0.06 µg/L and *P. aeruginosa* H103 at 0.06 µg/L, respectively. Only 11 function groups were used to build the SAR growth models due to the absence of “evident” significant descriptors.

From the 11 descriptors, SAR models were constructed as regressions based on multivariate analysis of variance (ANOVA). The ANOVA models/equations obtained for the prediction of the growth of the three strains of bacteria are presented in Table 2.

**Figure 8:**
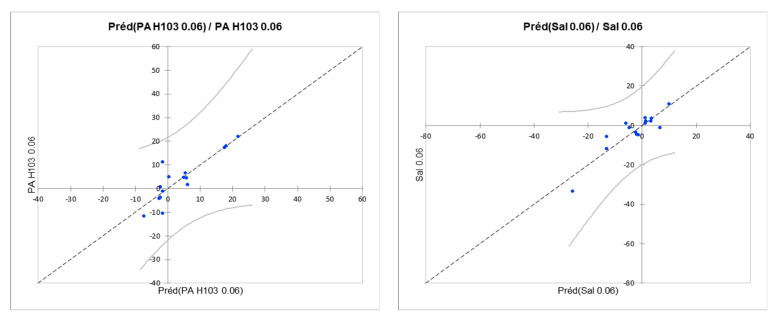
Comparison of the values predicted by the ANOVA model with the experimental values obtained for the growth of *P. aeruginosa* H103 at 0.06 µg/L and *S.* Typhimurium at 0.06 µg/L.

The fit coefficients (Table 2) showed that the models created failed to satisfactorily model the growth of *E. coli* K12 (at 0.06 and 0.3 µg/L), *P. aeruginosa* H103 (at 0.3 µg/L), and *S.* Typhimurium (at 0.3 µg/L). In contrast, the models obtained for *P. aeruginosa* H103 and *S.* Typhimurium at 0.06 µg/L were more significant (better R^2^, better adjusted R^2^, and lower RMSE [root mean square error]). A view of the predictive quality of these models is represented by the comparison of the values predicted by the model with the experimental values (Figure 8);

(c) SAR model for bacteria swarming

**Figure 9:**
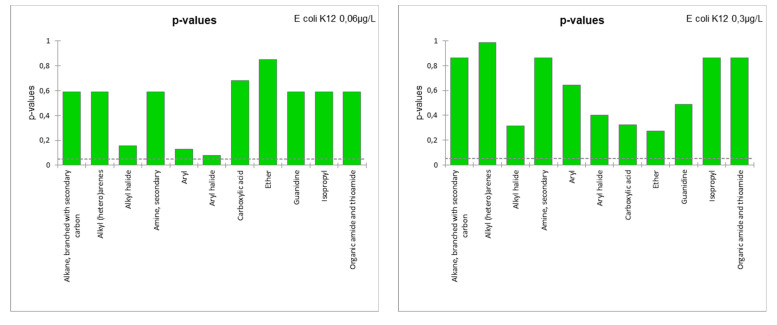

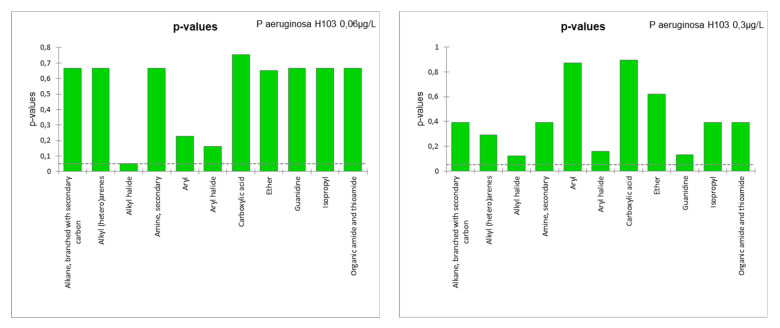
*p*-values of the Fisher F-test for the functional groups used as descriptors of swarming data.

Another SAR model was also built for predicting the swarming data by the same approach. The Fisher F-test results (Figure 9) show that the p-values of the different groups were all above the significance level for all swarming data. Some values were close to the 95% threshold (e.g., alkyl halide for *P. aeruginosa* H103 at 0.06 µg/L). Note that no model was built for S. Typhimurium, as the results of swarming did not show any conclusive results (Figure 2). SAR models were constructed, but their fit coefficients show that these models were not able to reliably predict the data (Table 3).

(d) Interaction between function groups

An important limitation of the models established above is the consideration of function groups independently of each other. However, the association of several groups can strongly control the physicochemical properties of a chemical structure and explain, amplify, or inhibit its biological effect. Indeed, function groups can interact and influence each other. Thus, the multivariate ANOVA models were extended by adding the possibility of interaction between the different functional groups. In total, 185 interactions—1 × 1 between function groups—were identified and added as potential descriptors that could be used to build the models. Model and fit coefficients are presented in Table 4.

**Figure 10:**
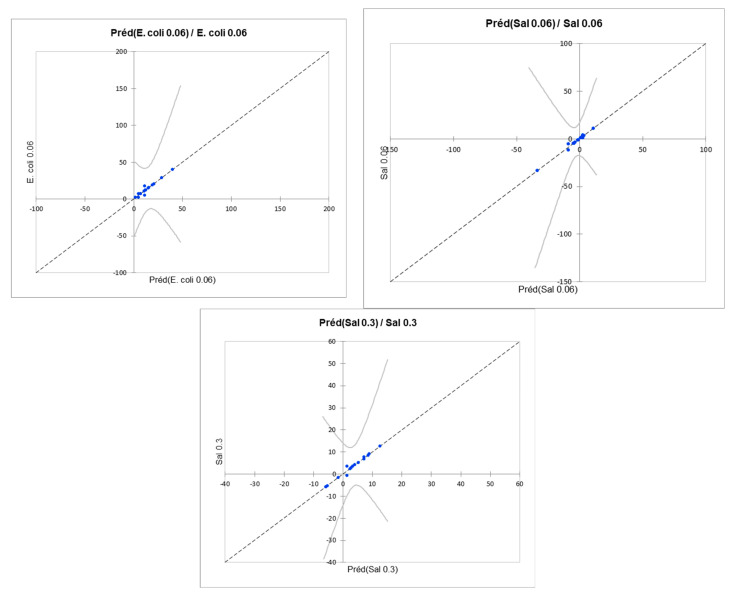
Comparison of the values predicted by the ANOVA model with the experimental values obtained for the growth of *E. coli* at 0.06 µg/L and *S.* Typhimurium at 0.06 µg/L and 0.3 µg/L.

The quality of fit showed that taking interactions into account allowed for a better representation of the experimental data from growth tests. Indeed, these models predicted growth with a better significance, especially for *E. coli* K12 and *S.* Typhimurium at 0.06 or 0.3 µg/L. Figure 10 shows that the predicted data overlap well with the experimental data. It should be noted that the confidence interval was more uncertain for the ranges in the experimental domain, where there were fewer results.

The models with interactions are not, however, systematically more efficient than the simple model in predicting all data. Thus, the results of the fitting coefficients showed that they failed to describe the growth of *P. aeruginosa* H103. Similarly, these models appeared limited in predicting the swarming data (except for *P. aeruginosa* H103 at 0.3 µg/L) (Table 5). Furthermore, the addition of new variables (i.e., the n interactions) decreased the degree of freedom of the model which—in the context of a limited data set such as ours—can be a limitation when there is little variation/variance between data, as was the case for the swarming results (Table 5).

## 4. Conclusions

The current experimental results show and confirm that the exposure of bacteria to an organic chemical compound can result in a modification of their growth or their mobility. The effect is dependent on the exposure concentration and is more or less intense depending on each compound and each bacterium. Nevertheless, the lowest concentrations studied (i.e., 0.06 and 0.3 µg/L) seem to generate the most significant effects overall.

The use of modeling showed that the differences between the compounds’ activities seemed to be explained by taking into account the chemical structure. Indeed, QSAR models—built on the basis of the organic functions found as descriptors—appeared to be capable of correctly predicting the activity of the compounds on the bacteria. Thus, it appears possible to predict the effect of the compounds studied (at the concentrations studied: 0.06 and 0.3 µg/L) on the growth or mobility of the bacteria. The various QSAR modelling tests showed that the models were even better when they included interactions between groups of functions. However, models should only be used for making predictions for those chemicals that fall within the applicability domain of the model. It is also important to keep in mind that the data used to build the model do not explore all possibilities of the experimental domain and, therefore, the models have a large uncertainty in the ranges where there are no data. Furthermore, our models also suffered from the limited number of compounds used for training (i.e., construction). Indeed, the database should be expanded with compounds of similar and different structures to make the model more robust and universal. Similarly, it would be important to add compounds to enable internal and external validation of QSAR models. All chosen compounds were used to build the models without keeping any data for validation because of the limited compound base.

This first QSAR modelling approach shows, however, that it would be possible to predict the increase in virulence factors (such as growth or motility) linked to exposure to organic compounds and, thus, to use models to screen and identify “a priori” the compounds most at risk. This study offers intriguing possibilities for the development of QSAR-based models that can pre-identify compounds based on their chemical structure, with potential to impact virulence factors in bacteria. Given the vast number of chemical compounds utilized in daily life and industry, effective risk assessment and determination can be a complex challenge. Thus, continuing research into QSAR models and other artificial intelligence-based modeling approaches can help health and environmental institutions prioritize compounds for regulation.

## Figures and Tables

**Table 1:** Occurrence of organic functions found by the algorithm in the model compound.

Variable	Presence	Count
Alkane, branched with secondary carbon	no	13
	yes	2
Alkyl (hetero)arenes	no	13
	yes	2
Alkyl halide	no	12
	yes	3
Amine, secondary	no	13
	yes	2
Aryl	no	8
	yes	7
Aryl halide	no	9
	yes	6
Carboxylic acid	no	11
	yes	4
Ether	no	10
	yes	5
Guanidine	no	13
	yes	2
Isopropyl	no	13
	yes	2
Organic amide and thioamide	no	13
	yes	2

**Table 2:** SAR model for bacteria growth and fit coefficients (significant values are designated in bold).

Bacteria (Concentration)	R²	R² Adjusted	RMSE	Model
*E. coli* K12 (0.06 µg/L)	0.635	-0.021	10.218	*E. coli* 0.06 = 50.45–8.03*Alkane, branched with secondary carbon-non-19.8*Alkyl (hetero)arenes-non-2.10*Alkyl halide-non-6.87*Aryl-non+3.35*Aryl halide-non-1.79*Carboxylic acid-non-0.26*Ether-non-21.11*Guanidine-non+12.98*Organic amide and thioamide-non
*E. coli* K12 (0.3 µg/L)	0.353	-0.813	9.715	*E. coli* 0.3 = 35.59–1.17*Alkane, branched with secondary carbon-non-9.19*Alkyl (hetero)arenes-non+0.78*Alkyl halide-non-6.02*Aryl-non+4.90*Aryl halide-non-3.46*Carboxylic acid-non-3.53*Ether-non-3.22*Guanidine-non+9.44*Organic amide and thioamide-non
*P. aeruginosa* H103 (0.06 µg/L)	**0.769**	**0.352**	**7.985**	PA H103 0.06 = 42.28–0.45*Alkane, branched with secondary carbon-non-22.99*Alkyl (hetero)arenes-non+0.65*Alkyl halide-non-5.78*Aryl-non+6.15*Aryl halide-non-13.82*Carboxylic acid-non+11.52*Ether-non-7.34*Guanidine-non-11.81*Organic amide and thioamide-non
*P. aeruginosa* H103 (0.3 µg/L)	0.497	-0.409	8.433	PA H103 0.3 = 28.98–6.65*Alkane, branched with secondary carbon-non-10.88*Alkyl (hetero)arenes-non-4.13*Alkyl halide-non-1.87*Aryl-non+2.75*Aryl halide-non-7.36*Carboxylic acid-non+5.07*Ether-non-14.48*Guanidine-non+0.54*Organic amide and thioamide-non
*S.* Typhimurium (0.06 µg/L)	**0.806**	**0.456**	**7.377**	Sal 0.06 = 43.85–13.97*Alkane, branched with secondary carbon-non-28.87*Alkyl (hetero)arenes-non-14.33*Alkyl halide-non-12.95*Aryl-non+16.17*Aryl halide-non-3.71*Carboxylic acid-non-6.78*Ether-non-11.78*Guanidine-non+19.21*Organic amide and thioamide-non
*S.* Typhimurium (0.3 µg/L)	0.441	-0.564	6.498	Sal 0.3 = 7.53–0.30*Alkane, branched with secondary carbon-non-4.85*Alkyl (hetero)arenes-non-0.29*Alkyl halide-non-4.39*Aryl-non+5.09*Aryl halide-non+3.11*Carboxylic acid-non-1.70*Ether-non-5.02*Guanidine-non+3.65*Organic amide and thioamide-non

**Table 3:** SAR model for bacteria swarming and fit coefficients.

Bacteria (Concentration)	R²	R² Adjusted	RMSE	Model
*E. coli* K12 (0.06 µg/L)	0.672	0.082	5.052	*E. coli* 0.06 = −0.3–8.37*Alkane, branched with secondary carbon-non-5.01*Alkyl (hetero)arenes-non+4.18*Alkyl halide-non+3.35*Aryl-non+6.69*Aryl halide-non-1.68*Carboxylic acid-non+1.66*Ether-non-3.35*Guanidine-non+5.02*Organic amide and thioamide-non
*E. coli* K12 (0.3 µg/L)	0.779	0.382	34.721	*E. coli* 0.3 = −259.09 + 46.54*Alkane, branched with secondary carbon-non-1.24*Alkyl (hetero)arenes-non+72.54*Alkyl halide-non-17.59*Aryl-non+6.10*Aryl halide-non+88.94*Carboxylic acid-non-40.25*Ether-non+71.79*Guanidine-non+76.55*Organic amide and thioamide-non
*P. aeruginosa* H103 (0.06 µg/L)	0.655	0.035	8.971	PA 0.06 = −2.31–13.47*Alkane, branched with secondary carbon-non-0.58*Alkyl (hetero)arenes-non+7.94*Alkyl halide-non+8.04*Aryl-non+10.05*Aryl halide-non-5.83*Carboxylic acid-non-3.83*Ether-non-6.83*Guanidine-non+9.04*Organic amide and thioamide-non
*P. aeruginosa* H103 (0.3 µg/L)	0.709	0.185	20.124	PA 0.3 = 22.28–6.61*Alkane, branched with secondary carbon-non-13.40*Alkyl (hetero)arenes-non-23.82*Alkyl halide-non+1.76*Aryl-non-26.65*Aryl halide-non+8.00*Carboxylic acid-non+3.19*Ether-non+20.51*Guanidine-non-21.29*Organic amide and thioamide-non

**Table 4:** SAR model with interactions for bacteria growth and fit coefficients (significant values are designated in bold).

Bacteria (Concentration)	R²	R² Adjusted	RMSE	Model with Interactions
*E. coli* K12 (0.06 µg/L)	**0.941**	**0.586**	**6.509**	*E. coli* 0.06 = 2.30+10.49*Alkane, branched with secondary carbon-non-25.59*Alkyl (hetero)arenes-non+18.39*Alkyl halide-non+25.94*Aryl-non+16.99*Aryl halide-non+14.39*Carboxylic acid-non+8.99*Ether-non-14.40*Guanidine-non+10.79*Organic amide and thioamide-non-12.69*Alkane, branched with secondary carbon-non*Aryl halide-non-24.94*Alkyl halide-non*Aryl-non-25.90*Aryl-non*Aryl halide-non
*E. coli* K12 (0.3 µg/L)	0.910	0.369	5.731	*E.coli* 0.3 = −22.74+21.99*Alkane, branched with secondary carbon-non-19.94*Alkyl (hetero)arenes-non+30.99*Alkyl halide-non+24.84*Aryl-non+20.94*Aryl halide-non+10.84*Carboxylic acid-non+8.04*Ether-non+7.19*Guanidine-non+10.09*Organic amide and thioamide-non-22.99*Alkane, branched with secondary carbon-non*Aryl halide-non-35.09*Alkyl halide-non*Aryl-non-10.5*Aryl-non*Aryl halide-non
*P. aeruginosa* H103 (0.06 µg/L)	0.823	-0.238	11.039	PA H103 0.06 = 33.90+3.69*Alkane, branched with secondary carbon-non-29.09*Alkyl (hetero)arenes-non+6.69*Alkyl halide-non-12.08*Aryl-non+13.29*Aryl halide-non-16.1*Carboxylic acid-non+13.59*Ether-non-4.10*Guanidine-non-6.10*Organic amide and thioamide-non-13.89*Alkane, branched with secondary carbon-non*Aryl halide-non-4.81*Alkyl halide-non*Aryl-non+15.39*Aryl-non*Aryl halide-non
*P. aeruginosa* H103 (0.3 µg/L)	0.685	-1.202	10.543	PA H103 0.3 = −10.72+9.94*Alkane, branched with secondary carbon-non-22.77*Alkyl (hetero)arenes-non+14.04*Alkyl halide-non+7.97*Aryl-non+23.42*Aryl halide-non-0.32*Carboxylic acid-non+13.37*Ether-non-6.45*Guanidine-non+8.24*Organic amide and thioamide-non-27.89*Alkane, branched with secondary carbon-non*Aryl halide-non-18.34*Alkyl halide-non*Aryl-non+3.24*Aryl-non*Aryl halide-non
*S.* Typhimurium (0.06 µg/L)	**0.983**	**0.882**	**3.434**	Sal 0.06 = 17.22–1.55*Alkane, branched with secondary carbon-non-43.92*Alkyl (hetero)arenes-non-0.35*Alkyl halide-non-23.09*Aryl-non+38.57*Aryl halide-non-5.37*Carboxylic acid-non-0.57*Ether-non-3.85*Guanidine-non+33.44*Organic amide and thioamide-non-35.49*Alkane, branched with secondary carbon-non*Aryl halide-non-10.83*Alkyl halide-non*Aryl-non+27.04*Aryl-non*Aryl halide-non
*S.* Typhimurium (0.3 µg/L)	**0.978**	**0.847**	**2.033**	Sal 0.3 = −10.65+6.39*Alkane, branched with secondary carbon-non-5.35*Alkyl (hetero)arenes-non+17.39*Alkyl halide-non+12.88*Aryl-non-4.14*Aryl halide-non+6.54*Carboxylic acid-non+1.65*Ether-non-1.00*Guanidine-non-3.39*Organic amide and thioamide-non+3.19*Alkane, branched with secondary carbon-non*Aryl halide-non-23.23*Alkyl halide-non*Aryl-non+1.10*Aryl-non*Aryl halide-non

**Table 5:** SAR model with interactions for bacteria swarming and fit coefficients.

Bacteria (Concentration)	R²	R² Adjusted	RMSE	Model with Interactions
*E. coli* K12 (0.06 µg/L)				The variance between the data is not significant enough
*E. coli* K12 (0.3 µg/L)	0.816	-0.288	50.138	*E.coli* 0.3 = −253.27+46.49*Alkane, branched with secondary carbon-non-12.62*Alkyl (hetero)arenes-non+46.5*Alkyl halide-non-52.74*Aryl-non+50.92*Aryl halide-non+86.77*Carboxylic acid-non-40.27*Ether-non+69.99*Guanidine-non+102.9*Organic amide and thioamide-non-37.69*Alkane, branched with secondary carbon-non*Aryl halide-non+40.11*Alkyl halide-non*Aryl-non+2.90*Aryl-non*Aryl halide-non
*P. aeruginosa* H103 (0.06 µg/L)				The variance between the data is not significant enough
*P. aeruginosa* H103 (0.3 µg/L)	0.950	0.651	13.164	PA 0.3 = −17.15+11.81*Alkane, branched with secondary carbon-non-35.90*Alkyl (hetero)arenes-non+11.81*Alkyl halide-non-12.27*Aryl-non-11.07*Aryl halide-non-0.59*Carboxylic acid-non+12.40*Ether-non+35.31*Guanidine-non-5.88*Organic amide and thioamide-non-47.13*Alkane, branched with secondary carbon-non*Aryl halide-non-35.33*Alkyl halide-non*Aryl-non+64.61*Aryl-non*Aryl halide-non

## Data Availability

All data included in this study are available upon request by contacting the corresponding author.

## Supplementary Materials

The following supporting information can be downloaded at: https://www.mdpi.com/article/10.3390/microorganisms11061375/s1, Table S1: List of pesticides use as model compounds; Table S2: List of pharmaceuticals use as model compounds; Table S3: Physicochemical properties of model compounds; Table S4: Primer pair used for virulence characterization of *P. aeruginosa* H103 and *S.* Typhimurium isolates; Figure S1: Percentage of bacteria growth in the presence of MeOH.
