# Supplementary material for: Application of QSAR Approach to Assess the Effects of Organic Pollutants on Bacterial Virulence Factors
Source: Microorganisms. 2023 May 24;11(6):1375. doi: 10.3390/microorganisms11061375 (PMC10301662; doi:10.3390/microorganisms11061375)
Supplement: Supplementary file 1 [file microorganisms-11-01375-s001.zip › microorganisms-2367273-supplementary.pdf]

Supporting information

Application of QSAR Approach to Assess the Effects of Organic Pollutants on Bacterial Virulence Factors

Roukaya Al Haj Ishak Al Ali [1], Leslie Mondamert [1], Jean-Marc Berjeaud [2], Joelle Jandry [3], Alexandre Crépin [2] and Jérôme Labanowski [1,*]

[1] Institute of Chemistry, Materials and Natural Resources of Poitiers, UMR CNRS 7285, University of Poitiers, 86000 Poitiers, France; roukaya.ishak@hotmail.com (R.A.H.I.A.A.); leslie.mondamert@univ-poitiers.fr (L.M.)
[2] Ecology and Biology of Interactions, UMR CNRS 7267, University of Poitiers, 86000 Poitiers, France
[3] Faculty of Agronomy and Veterinary Sciences, Lebanese University, Dekwaneh, Lebanon
* Correspondence: jerome.labanowski@univ-poitiers.fr

Table S1. List of pesticides use as model compounds

**Organochlorinated compounds**		
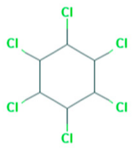 α-HCH, β-HCH, Lindane	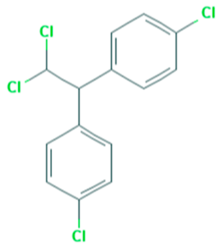 2,4’-DDD	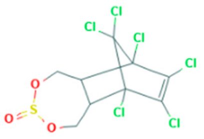 Endosulfan
**Organonitrogen/chlorine compounds**		
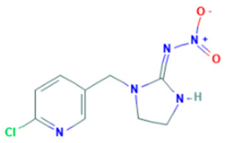 Imidacloprid	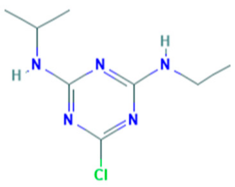 Atrazine	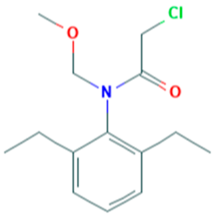 Alachlor
**Chlorophenoxy compounds**		
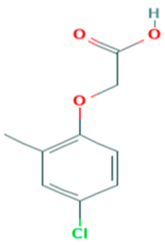 MCPA	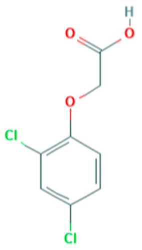 2,4-D	

Table S2. List of pharmaceuticals use as model compounds

**Anti-inflammatory compounds**		
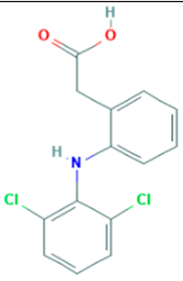 Diclofenac	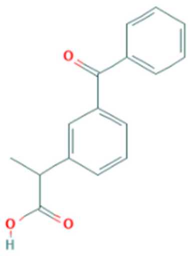 Ketoprofen	
**Other pharmaceutical compounds**		
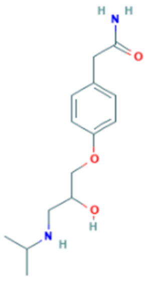 Atenolol	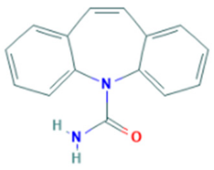 Carbamazepine	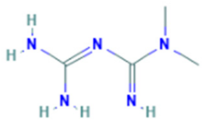 Metformin
**Antibiotics**		
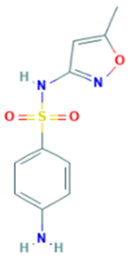 Sulfamethoxazole	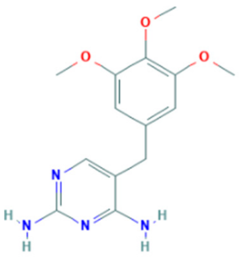 Trimethoprim	

Table S3. Physico-chemical properties of model compounds

Chemical name	MW	Log P	log Kow	Water Solubility	Koc	Ultimate biodeg	VdW volume	LUMO-HOMO energy
∑HCH	290.30	3.72	4.26	4.04	3.59	1.52	160	11.36
2,4'-DDD	320.04	6.02	5.87	0.07	5.22	1.66	206	9.25
Endosulfan	406.90	3.83	3.50	1.49	3.20	0.63	216	9.53
Imidacloprid	255.60	0.57	0.56	7170.00	1.53	2.21	171	9.31
Atrazine	215.68	2.61	2.82	214	2.16	2.00	163	9.49
Alachlor	269.76	3.52	3.37	18.10	2.73	2.22	212	9.57
MCPA	200.62	3.25	2.52	181	2.01	2.78	138	9.23
2,4-D	221.04	2.81	2.62	336	1.77	2.60	136	9.16
Atenolol	266.34	0.16	-0.03	685	0.61	2.61	216	9.27
Ketoprofene	254.28	3.12	3.00	120	2.08	2.93	194	9.44
Diclofenac	296.10	4.51	4.02	4.52	2.61	2.29	199	8.48
Metformine	129.16		-1.40	1.00E+06	0.04	2.91	107	9.92
Carbamazepine	236.27	2.45	2.25	17.70	2.23	2.68	178	8.19
Trimethoprim	290.32	0.91	0.73	2330.00	1.90	2.04	219	8.80
Sulfamethoxazole	253.28	0.89	0.48	3940.00	1.54	2.43	171	8.67

Table S4. Primer pair used for virulence characterization of *P. aeruginosa* H103 and *S.* Typhimurium isolates

Bacteria	Gene	Primer sequence (5'→3')	Annealing temperature (˚C)
***P. aeruginosa* H103**	*algD*	F: AGTACACCTGCAACGTCTGG<br>R: CATGTAGTAGCGCGACAGGT	58˚C
	lasI	F: GCGTGCTCAAGTGTTCAAGG<br>R: ATTCGCCAGCAACCGAAAAC	58˚C
	exoS	F: AGGTCGAGGAGTCCCGTATC<br>R: CAACTGGTCGGTGATTTCGC	56˚C
	oprL	F: TTCCTGTGTATGACGGCGTT<br>R: GAGCTGCATGAACAGTTCGC	51˚C
***S.* Typhimurium**	*invA*	F: ACGTTTCCTGCGGTACTGTT<br>R: GTGGCGATAATTTCACCGGC	58˚C
	stn	F: GCGGTCAGTCCCACTTTCTT<br>R: CGCTTACGCCTATTCAGGCT	56˚C
	rss16S	F: AAACGGTGGCTAATACCGCA<br>R: GAGCCGTTACCTCACCAACA	60˚C

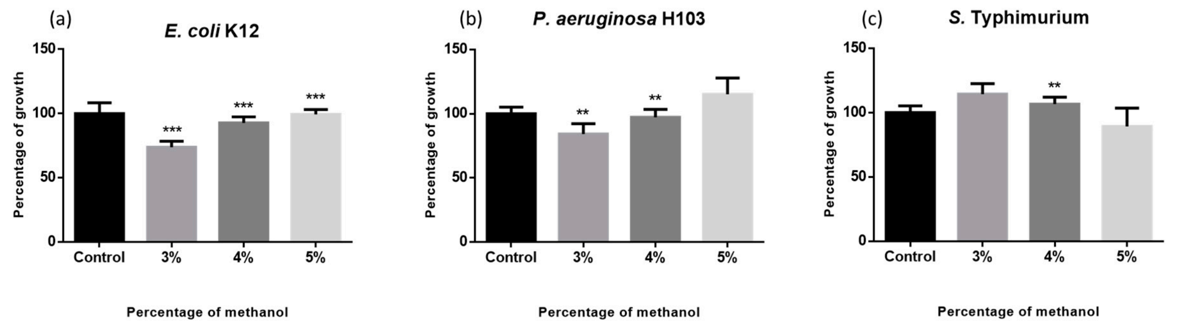

Figure S1. Percentage of bacteria growth in the presence of MeOH. (a) *E. coli* K12, (b) *P. aeruginosa* H103, (c) *S.* Typhimurium. Results were subjected to unpaired (independent) parametric T-test (GraphPad Prism version 6.01 for Windows, GraphPad Software, La Jolla California USA, www.graphpad.com). For qPCR tests, statistical analyses were performed on ΔCt values. All test's results were significant when p values were < 0.05 (*p< 0.05; **p< 0.01; ***p < 0.001)
